# Public oral health services performance in Brazil: Influence of the work process and service structure

**DOI:** 10.1371/journal.pone.0233604

**Published:** 2020-05-29

**Authors:** Leonardo de Paula Amorim, Maria Inês Barreiros Senna, Gizelton Pereira Alencar, Lorrany Gabriela Rodrigues, Janice Simpson de Paula, Raquel Conceição Ferreira

**Affiliations:** 1 Department of Social and Preventive Dentistry, School of Dentistry, Universidade Federal de Minas Gerais, Belo Horizonte, Minas Gerais, Brazil; 2 Department of Dental Clinic, Pathology and Surgery, School of Dentistry, Universidade Federal de Minas Gerais, Belo Horizonte, Minas Gerais, Brazil; 3 Department of Epidemiology, School of Public Health, Universidade de São Paulo, São Paulo, São Paulo, Brazil; Centre Hospitalier Regional Universitaire de Tours, FRANCE

## Abstract

The quality of oral health care might be evaluated based on Donabedian’s structure-process-outcome model. This study assessed the association between the oral health public services structure and work process of oral health teams (OHT) and performance indicators (access and problem-solving capacity) in Brazil. Secondary data from a national program obtained through interviews and by observation in 2013/2014 were analyzed. The performance indicators were Coverage of First Scheduled Dental Appointment (FDA) (< or ≥ the mean) and Ratio between Completed Treatments and First Scheduled Dental Appointments (CT/FDA) (< 1 or ≥ 1). The structure was assessed by the sum of available instruments, equipment, and supplies. Latent class analyses were used to identify similar groups (consolidated, developing, and incipient) of OHT according to the work process (planning of actions, health promotion and intersectoral actions, and integral health care). Each OHT was also described regarding the number of the health team in which the OHT operates, whether the primary care unit receives students/teaches, frequency of care provided outside of OHT coverage, and participation in telehealth. Multiple logistic regression models were adjusted for each performance indicator. A total of 16189 (99,8%) and 16192 (99,9%) OHTs located in 4344 (78,0%) municipalities had complete data on the work process and structure. 91.92% of OHTs presenting CT/FDA ≥ 1 and 37.05% presenting FDA ≥ the mean. Consolidated planning of actions and better structural conditions were associated with better performance. A higher frequency of CT/FDA ≥ 1 was observed among OHTs with consolidated integral health care and those that performed telehealth. OHTs that served individuals outside of OHT coverage daily and that worked with two to nine Health Teams presented a higher frequency of FDA ≥ the mean. OHTs with better structural and work process conditions had better performance.

## Introduction

The Unified Health System created by the 1988 Brazilian Federal Constitution ensures universal and integral health care to the population [1]. The family health strategy is a model aimed at reorganizing primary health care with an emphasis on teamwork. The National Oral Health Policy, implemented in 2004, boosted the inclusion of oral health teams (OHT—dentist, dental technician, and assistant) integrated with the Family health team (FHT–physician, nurse, community health agents, technician, and nursing assistant) in primary care units, expanding the access of the Brazilian population to oral health services [2, 3]. The coverage of oral health care increased from 20.5% in 2003 [4] to 46.2% in 2007, followed by a slower progression, reaching 52.5% in March 2019 [5].

The National Oral Health Policy assumes that the expansion and qualification of care enable effective problem solving, ensuring integral health care with continuous evaluation practices involving the use of health indicators [2]. The institutionalization of the evaluation of primary care quality was strengthened in 2011 with the implementation of the National Program for Improvements in Primary Care Access and Quality (PMAQ-AB) [6], which include evaluation of the OHTs.

The PMAQ-AB is based on Donabedian’s structure-process-outcome model with strategies for qualification, monitoring, and evaluation of health teams linked to a financial incentive to municipalities that meet the standards of access and quality. It is organized into biennial cycles developed in continuous and complementary phases [6]. The team's adhesion to the program occurs through agreements of commitments and performance indicators. The OHT performance indicators include measurements of access and problem-solving capacity. The access indicators capture the opportunity of the population to get oral health care and may point to the insertion of oral actions in health programs, such as mental health, women, workers, and adolescents. The problem-solving capacity indicators reveal the performance of the services to solve the oral health needs presented by users [7]. Both indicators might guide changes in the care services supporting the planning and management of oral health actions [8, 9].

The PMAQ-AB results can reveal the need for investments in structure or motivate teams to think about the work process, contributing to change in the way care is provided and managed, and thus enable better-qualified actions and services. The relationship between the work process, structure, and performance indicators may highlight priority aspects to be considered by local and national managers and health professionals aiming to improve the quality of the service. The previous studies have shown that despite the remarkable advances in public health policies, challenges, and obstacles persist, including difficulties on integration between OHT and FHT [10] and structural needs [11]. The Process-structure-outcomes model is widely used in service evaluation studies. However, most studies have been focused on one or two components of evaluation, for example, process and structure [8, 11, 12]. According to this theoretical model, structure measures have an effect on process measures, which in turn affect outcome measures [13]. Although this is a complex causal relationship, the advancing to understand this association, considering a selection of relevant measures, seems an innovative aspect of this study.

In the context of the expansion of the OHT in Brazil with a consequent need for evaluation of the quality of provided services, this study aimed to evaluate the association between structure and work process quality and performance of OHTs considering indicators of access and problem-solving capacity at Brazilian public oral health services.

## Materials and methods

### Study design, population, and sample

An analytical study conducted using secondary data whose unit of analysis was the OHT at primary care units of the Unified Health System. The sample was all OHT that adhered to the second cycle of the PMAQ-AB conducted in 2013/2014.

Two databases were accessed for this study. The database containing the performance indicators for each OHT in 2014, identified by the National Registry of Health Establishments, was made available to the researchers by the Health Ministry upon presentation of the research project approved by the ethics committee. The National Registry of Health Establishments is the basis for operationalizing Brazilian health information systems [14]. This request to the Health Ministry was necessary because, on public databases, the indicators were aggregated by the municipality, making it impossible to assume the OHT as a unit of analysis.

The PMAQ-AB database was available at the websites of the Primary Care Department of the Health Ministry [15]. This database was organized into six modules, and modules V and VI were used in this study. Data on the structure of health services, obtained from observations in primary care units, were extracted from module V. The work process was evaluated by an interview with one of the OHT members (dentist, dental technician or oral health assistant), and data were obtained from module VI. A team of evaluators without a link to the service, which had previously undergone training at education and research institutions, conducted the interviews and observations [6].

### Evaluation of access and problem-solving capacity of oral health services

Access and problem-solving capacity were assessed based on performance indicators of oral health services: Coverage of First Scheduled Dental Appointments (FDA) and the Ratio between Completed Treatments and First Scheduled Dental Appointments (CT/FDA). These two indicators are calculated based on regular dental consultations registered in the national information system by OHT. The FDA indicator aims to measure the proportion of individuals who had access to dental treatment by the OHT. The calculus equation is the ratio between the total number of FDAs performed by the OHT in 2014 and the population registered in the OHT in the same year. Sporadic consultations, such as cases of urgent care and appointments with no expected continuity, were not considered in this calculation [7]. Higher percentage values indicate greater access. The analysis of this indicator was based on the parameter from the Health Ministry for the classification of OHT performance: OHTs with fair performance (< mean) and those with good and excellent performance (≥ mean).

The CT/FDA ratio enables assessing whether the OHT maintains a good balance between access (number of first scheduled dental appointments) and problem-solving capacity (number of treatments completed), indicating the extent to which the team completes the initiated treatments. The indicator results from the ratio between the total number of completed treatments by the OHT in the year divided by the number of FDAs performed by the OHT in the same year [7]. A ratio of 1 represents the ideal situation. For the analysis, the indicator was dichotomized as < 1 or ≥ 1. Values < 1 indicate that the number of completed treatments is lower than the number of initiated treatments, suggesting a possible low problem-solving capacity. A result ≥ 1 indicates that treatments are being completed without new treatments being initiated, showing a possible problem with access to oral health services for new patients.

### Assessment of oral health team structure

The structure of the OHT was assessed based on the availability (code 1) or unavailability (code 0) of a set of equipment, instruments, and supplies considered necessary for clinical dental care. For analysis, a methodology adapted from Queiroz *et al* [16] was adopted, obtaining the sum of all available items for each aspect of the structure.

The sum of equipment could result in values from 0 to 9 according to availability of a dental office at the primary care unit, dental chair, compressor, vehicle available for activities outside the primary care unit, adjustable office chair, reflector, cuspidor, aspirator, and light-curing device. The supplies were acid/adhesive, cotton rolls, vasoconstricting anesthetics, drills, dental cements, personal protective equipment (gloves, masks, aprons, and caps), fluoride gel, gauze, temporary restorative material, carbon paper, sealants, light-curing resins, and needle disposal box, totaling 0 to 13. The instruments were calcium hydroxide applicator, micro brush, resin insertion spatula, clinical forceps, mouth mirror, scanning probe, glass plate, and carpule syringe, totaling 0 to 8.

### Assessment of the work process of OHT

The PMAQ-AB included a large number of variables regarding the planning of actions; health promotion and intersectoral cooperation actions and integral health care (Table 1). Three separate latent class analyses (LCA) were performed for each set of observed variables because they measured different aspects of the work process. LCA was employed to identify OHT similar subgroups (classes) of OHT according to profile for this set of variables. LCA is a mixed model that postulates the existence of an underlying and unobserved categorical variable (latent variable) that divides a population into mutually exclusive and complete latent classes. The participation of individuals in the categories is unknown but can be inferred from measuring a set of items [17].

**Table 1:** Observed work process variables included in latent class analysis.

Variables	Code in the PMAQ-AB database	Categories*
***Planning of Actions (9 items)***		
Oral Health Team plans/programs activities weekly, biweekly or monthly.	VI.9.2.1	0"no"1"yes”
Oral Health Team plans/programs activities considering primary care goals determined by the municipality.	VI.9.2.2	0"no"1"yes”
Oral Health Team plans/programs activities considering information from the primary care information system.	VI.9.2.3	0"no"1"yes”
Oral Health Team plans/programs activities considering local information (demand study, epidemiological scenario, and others).	VI.9.2.4	0"no"1"yes”
Oral Health Team plans/programs activities considering issues related to biological risks and individual, family, and social vulnerabilities (violence, drugs, and others).	VI.9.2.5	0"no"1"yes”
Oral Health Team plans/programs activities considering environmental issues of an area of coverage (including access to land).	VI.9.2.6	0"no"1"yes”
Oral Health Team plans/programs activities considering challenges identified in self-evaluation.	VI.9.2.7	0"no"1"yes”
Does Oral Health Team monitor and analyze oral health indicators and information?	VI.7.2	0"no"1"yes”
Does management make available to Oral Health Team information that assists in health situation analysis?	VI.7.4	0"no"1"yes”
***Health Promotion and Intersectoral Actions (4 items)***		
Is Oral Health Team agenda organized to offer oral health education activities in the area of coverage?	VI.13.5	0"no"1"yes”
Does Oral Health Team follow up pregnant women through appointments?	VI.18.1	0"no"1"yes”
Does Oral Health Team conduct home visits?	VI.20.1	0"no"1"yes”
Does Oral Health Team carry out activities school/daycare center?	VI.22.1	0"no"1"yes”
***Integral Health Care (3 items)***		
Is central regulation available to refer users to dental specialties?	VI.15.1	0"no"1"yes”
Does Oral Health Team offer specialized consultations available from health network?	VI.14.1	0"no"1"yes”
Does Oral Health Team carry out actions to prevent and detect oral cancer?	VI.17.3	0"no"1"yes”

*The answer Yes was the most favorable or positive one.

A sequence of models for the set of observed variables, containing from one to five classes, was tested to determine the best result based on the minimum value of the BIC (Bayesian Information Criterion). Then, for each latent variable, the probability of each OHT belonging to a subgroup (classes) was estimated. From the maximum probability values, the distribution of the OHT in one of the classes was defined. For interpretation purposes, the classes represented the different OHT performances in terms of the work process. OHT with a high probability of favorable answers to the observed questions were grouped into a class called “consolidated work process” and those with a lower probability called “work process under development or incipient.” The LCA was performed using the generalized structural equation model with the logit function, considering that all observed variables were binary.

The evaluation of OHT work process also included the followings variables: 1) the number of FHTs in which OHT operates (1; 2 to 9), 2) receiving students/teachers in teaching activities (no; 1 to 6 times a week), 3) frequency of care provided outside of OHT coverage area (no; some days of the week; every day of the week), and 4) participation of OHT in telehealth (yes; no). The National Telehealth Brazil Program was implemented in 2007 and seeks to improve the quality of primary care by offering actions aimed at health professionals through information and communication technologies, namely Teleconsultations, Formative Second Opinion, Tele-Education and Tele-Diagnosis [18].

### Professional profiles

The OHT professionals were characterized with regards to the conclusion of complementary training (yes, no), employment (statutory public servant or hired public employee; contract services, temporary contract, commissioned position, self-employed or others), work regimen with a career path (yes, no). The interviewed professional was a dentist to 94.5% of the OHT.

### Statistical analysis

PMAQ-AB databases (modules V and VI) and performance indicator databases were linked using the number of National Registry of Health Establishments. This number identifies each primary care unit, and it was the same in the three databases. For module VI, although only one OHT professional should be interviewed, there were cases in which two or more professionals responded to the same team. In cases of disagreement, we opted for the most positive response to obtain the data for the team. This situation occurred to all variables regarding the work process.

**Fig 1:**
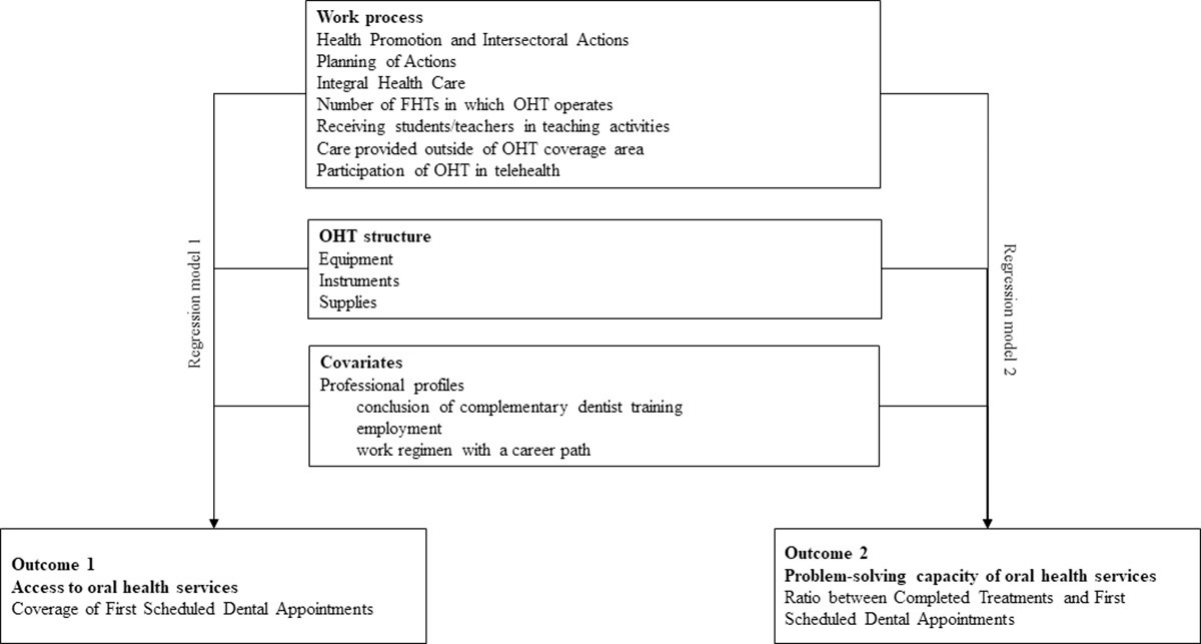
Flowchart of the variables included in regression models. OHT: Oral Health Team. FHT: Family Health Team.

Descriptive analysis was performed to characterize the OHTs regarding the performance indicators, work process and profile of the professionals. Two logistic regression models were adjusted to investigate the association of structure, work process and professional profile with each performance indicator (Coverage of First Scheduled Dental Appointments and the Ratio between Completed Treatments and First Scheduled Dental Appointments). The variables included in the analysis were presented in Fig 1. All variables associated with performance indicators with p < 0.20 in the crude model were incorporated into the multiple models. Pearson's chi-square and the Hosmer-Lemeshow chi-square tests were used to evaluate the goodness of fit of the model. The Stata 15 software was used for all statistical analyses.

### Ethical aspects

This study received approval from the Research Ethics Committee of the Federal University of Minas Gerais (certificate number: 76981917.4.0000.5149). Secondary public data were used without the identification of participants. The PMAQ-AB database is available on the Health Ministry website [15]. The adherence of health teams to the program was voluntary through the signing of a Term of Adherence.

## Results

**Fig 2:**
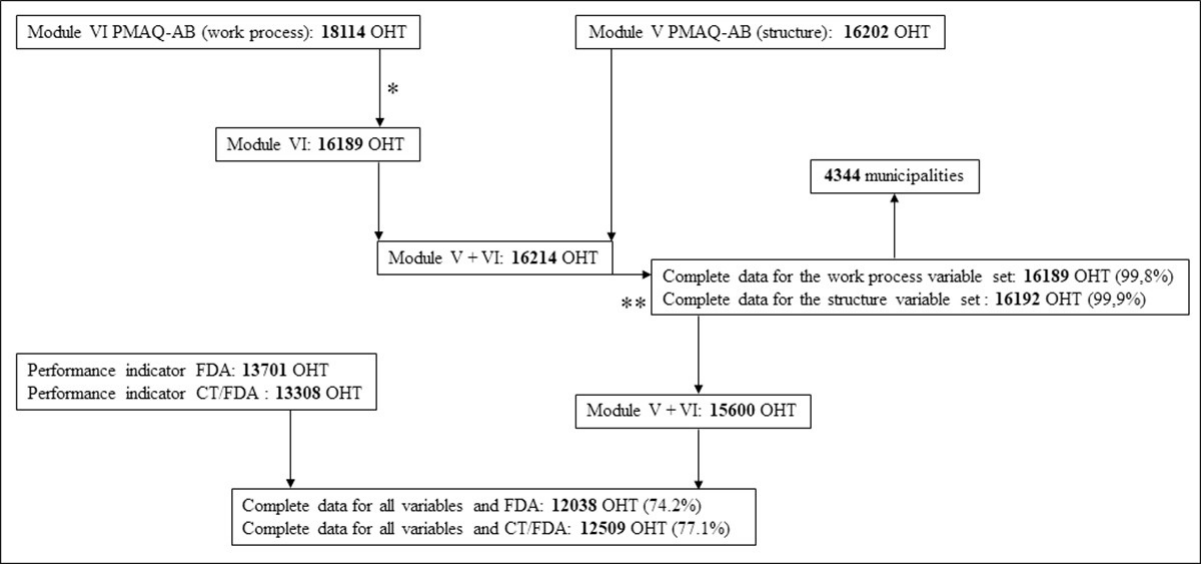
Flowchart of database linking steps. FDA: Coverage of First Scheduled Dental Appointments. CT/FDA: Ratio between Completed Treatments and First Scheduled Dental Appointments OHT: Oral Health Team. * Number after collapse of OHT data from the same primary care unit. ** Missing. Module VI: Database of PMAQ-AB with variables concerning work process of OHT Module V: Database of PMAQ-AB with variables concerning structure of OHT.

The flowchart (Fig 2) shows the steps involved in linking the databases and the number of participating teams.

### Characterization of the work process and structure of the oral health teams

A total of 16189 and 16192 OHTs had complete work process and structure data, corresponding to 99,8% and 99,9% of the teams participating in PMAQ-AB, respectively. These teams were located in 4344 municipalities (78,0% of all municipalities in Brazil).

Table 2 displays the distribution of the observed variables concerning the *planning of actions*, *health promotion and intersectoral actions*, and *integral health care*. For the planning of actions, the LCA resulted in three classes nominated as consolidated (OHT with more favorable features of planning of actions), developing (intermediate features of planning of actions), and incipient (fewer favorable features of planning of actions). Two classes were obtained for *health promotion and intersectoral actions* and *integral health care*, which were nominated as developing and consolidated according to less or more favorable features of the work process, respectively. The frequency of OHT with consolidated *planning of actions* was 59.09%. Consolidated *health promotion and intersectoral actions* were observed to 89.59% of the OHT, and 68.78% presented consolidated *integral health care*.

**Table 2:** Frequency of favorable responses* to observed variables on planning of actions; health promotion and intersectoral actions; integral health care that composed latent subgroups: Incipient, under development or consolidated.

Observed Variables	Action planning: incipient n = 1476 (%)	Action planning: under development n = 5147 (%)		Action planning: consolidated n = 9566 (%)	Total n = 16189 (%)
**Oral Health Team plans/programs activities weekly, biweekly or monthly.**	55.5	91.6		98.5	92.4
**Oral Health Team plans/programs activities considering primary care goals determined by municipality.**	31.2	76.5		99.1	85.8
**Oral Health Team plans/programs activities considering information from primary care information system.**	15.1	64.1		98.7	80.1
**Oral Health Team plans/programs activities considering local information (demand study, epidemiological scenario and others).**	3.7	68.3		98.9	80.5
**Oral Health Team plans/programs activities considering issues related to biological risks and individual, family and social vulnerabilities (violence, drugs and others).**	6.9	67.5		95.4	78.5
**Oral Health Team plans/programs activities considering environmental issues of area of coverage (including access to land).**	4.4	52.4		82.7	65.9
**Oral Health Team plans/programs activities considering challenges identified in self-evaluation.**	10.5	53.1		91.3	71.8
**Does Oral Health Team monitor and analyze oral health indicators and information?**	12.9	38.9		90.1	66.8
**Does management make available to Oral Health Team information that assists in health situation analysis?**	18.0	48.5		95.9	73.8
**Observed Variables**	**Health promotion and intersectoral actions: under development n = 1685 (%)**		**Health promotion and intersectoral actions: consolidated n = 14504 (%)**		
**Is Oral Health Team agenda organized to offer oral health education activities in area of coverage?**	35.4		97.1		90.7
**Does Oral Health Team follow up pregnant women through appointments?**	51.9		96.6		92.0
**Does Oral Health Team conduct home visits?**	13.6		85.5		78.0
**Does Oral Health Team carry out activities school/daycare center?**	49.4		97.7		92.7
**Observed Variables**	**Integral health care: under development n = 5055 (%)**		**Integral health care: consolidated n = 11134 (%)**		
**Is central regulation available to refer users to dental specialties?**	22.5		69.4		54.8
**Does Oral Health Team offer specialized consultations available from health network?**	15.9		100		73.8
**Does Oral Health Team carry out actions to prevent and detect oral cancer?**	56.6		90.8		80.1

*Favorable response was the Yes option for all questions.

The OHT, with consolidated *planning of actions*, used different sources of information on the area of coverage for planning actions, considered the risk and vulnerability factors, working with goals and indicators, performed self-evaluations, and created a periodic work schedule. The group with consolidated *health promotion and intersectoral actions* offered health education activities, including at schools and daycare centers, performed home visits, and monitored pregnant women. Likewise, the group with consolidated "integral health care" had a higher frequency of OHTs that performed oral cancer prevention and detection, offered specialized consultations, and had central regulation.

Regarding the structure, most OHTs had all equipment (58.79%), supplies (54.54%), and instruments (80.56%).

### Performance of oral health teams

A total of 13701 and 13308 OHTs were considered in the descriptive analyses of the FDA and CT/FDA indicators, respectively. More than 90% of the OHTs had a CT/FDA ratio ≥ 1 (91.92%), and 37.05% had FDA ≥ the mean.

### Association between work process and performance indicators of oral health services: First scheduled dental appointment and ratio between completed treatments and first scheduled dental appointments

The results of the bivariate analysis between the work process variables and performance indicators are presented in Tables 3 and 4. OHTs with incipient planning of actions and under development health promotion and intersectoral actions had a higher frequency of the coverage of the first scheduled appointment below the mean. The frequency of first scheduled appointment coverage ≥ mean was higher among OHTs that worked with 2 to 9 FHTs and served individuals outside their area of coverage every day of the week (Table 3).

**Table 3:** Bivariate analysis of *first scheduled dental appointment coverage* versus *work process* variables and *professional profiles*.

	First scheduled dental appointment coverage < mean		First scheduled dental appointment coverage ≥ mean		Total
	n	%	n	%	n
**Work process**					
**Planning of actions**					
Incipient	734	67.8	349	32.2	1083
Under development	2530	64.6	1388	35.4	3918
Consolidated	4492	62.1	2987	39.9	7479
**Health promotion and intersectoral actions**					
Under development	794	67.5	383	32.5	1177
Consolidated	6962	61.6	4341	38.4	11303
**Integral health care**					
Under development	2359	61.9	1453	38.1	3812
Consolidated	5397	62.3	3271	37.7	8668
**Participating of OHT in telehealth?**					
No	6163	62.4	3711	37.6	9874
Yes	1593	61.1	1013	38.9	2606
**Receiving students/teachers in teaching activities**					
No	6820	62.1	4157	37.9	10977
1 to 6 times/week	936	62.3	567	37.7	1503
**Number of FHTs in which OHT operates**					
1	1034	67.8	491	32.2	1525
2 to 9	6722	61.4	4233	38.6	10955
**Care to individuals outside of OHT coverage area**					
No	1024	64.5	563	35.5	1587
Some days of the week	4119	62.3	2489	37.7	6608
Every day of week	2613	61.0	1672	39.0	4285
**Professional profile**					
**Work regimen with a career path**					
No	6147	61.4	3868	38.6	10015
Yes	1386	65.4	734	34.6	2120
**Complementary dentist training**					
No	2319	61.8	1435	38.2	3754
Yes	5437	62.3	3289	37.7	8726
**Employment relationship**					
Contract services, temporary contract, commissioned position, self-employed or other	3745	60.4	2453	39.6	6198
A statutory public servant or hired public servant	3941	63.9	2231	36.1	6172

**Table 4:** Bivariate analysis of the *ratio between completed treatments and first scheduled dental appointment* versus *work process* variables and *professional profiles*.

	Ratio between completed treatments and first scheduled dental appointment < 1		Ratio between completed treatments and first scheduled dental appointment ≥ 1		Total
	n	%	n	%	n
**Work process**					
**Planning of actions**					
Incipient	122	11.3	957	88.7	1079
Under development	364	9.0	3660	91.0	4024
Consolidated	524	6.7	7324	93.3	7848
**Health promotion and intersectoral actions**					
Under development	133	11.6	1013	88.4	1146
Consolidated	877	7.4	10928	92.6	11805
**Integral health care**					
Under development	408	10.6	3447	89.4	3855
Consolidated	602	6.6	8494	93.4	9096
**Participation in telehealth**					
No	874	8.5	9372	91.5	10246
Yes	136	5.0	2569	95.0	2705
**Receiving students/teachers in teaching activities**					
No	895	7.9	10458	92.1	11353
1 to 6 times a week	115	7.2	1483	92.8	1598
**Number of FHTs in which OHT operates**					
1	107	6.7	1488	93.3	1595
2 to 9	903	8.0	10453	92.0	11356
**Care for individuals outside of OHT coverage area**					
No	137	8.4	1503	91.6	1640
Some days of the week	531	7.7	6328	92.3	6859
Every day of week	342	7.7	4110	92.3	4452
**Professional profile**					
**Work regimen with a career path**					
No	861	8.4	9434	91.6	10295
Yes	127	5.5	2187	94.5	2314
**Complementary dentist training**					
No	367	9.5	3486	90.5	3853
Yes	643	7.1	8455	92.9	9098
**Employment relationship**					
Contract services, temporary contract, commissioned position, self-employed or others	536	8.4	5878	91.6	6414
Statutory public servant or hired public servant	469	7.3	5961	92.7	6430

OHTs with a consolidated work process (planning of actions, health promotion and intersectoral actions and integral health care) had a lower frequency of CT/FDA < 1. Likewise, for the other variables, a favorable response contributed to the problem-solving capacity (Table 4).

The only OHTs without missing for all variables were included in regression models (model 1: 74.2%; model 2: 77.1%).

A higher chance of FDA ≥ mean was observed to OHTs with consolidated planning of actions compared to those with incipient planning of actions. The increase of one equipment was associated with a 13% more chance of coverage above the mean. OHTs served individuals outside their area of coverage every day of the week and worked with 2 to 9 FHTs showed a higher chance of FDA ≥ mean. When the municipality had a career plan, and the employment relationship of the professional was that of a statutory public servant or hired public employee, the chance of FDA coverage was lower (Table 5).

**Table 5:** Crude and adjusted regression models of the variables associated with first scheduled dental appointment coverage (FDA) ≥ mean.

	FDA ≥ mean	
Variables	Crude OR (95% CI)	Adjusted OR (95% CI)
**Work process**		
**Planning of actions**		
Incipient	1	1
Under development	**1.15(0.99–1.33)**	1.08 (0.93–1.26)
Consolidated	1.40(1.22–1.60)***	1.27(1.10–1.47)***
**Health promotion and intersectoral actions**		
Under development	1	
Consolidated	1.29(1.14–1.47)***	
**Integral health care**		
Under development	1	
Consolidated	0.98(0.91–1.06)	
**Participation of OHT in telehealth**		
No	1	
Yes	1.06(0.97–1.15)	
**Receiving students/teachers in teaching activities**		
No	1	
1 to 6 times a week	0.99(0.89–1.11)	
**Number of FHTs in which OHT operates**		
1	1	1
2 to 9	1.33(1.18–1.49)***	1.34(1.19–1.51)***
**Care for individuals outside of OHT coverage area**		
No	1	1
Some days of week	1.10(0.98–1.23)	1.07(0.96–1.21)
Every day of week	1.16(1.03–1.31)*	1.19(1.06–1.35)**
**Structure of OHT**		
**Equipment**	1.15(1.09–1.21)***	1.13(1.06–1.19)***
**Supplies**	1.05(1.03–1.08)***	
**Instruments**	1.06(1.01–1.11)*	
**Professional profile**		
**Work regimen with a career path**		
No	1	1
Yes	0.84(0.76–0.93)***	0.89(0.80–0.99)*
**Complementary dentist training**		
No	1	
Yes	0.98(0.90–1.06)	
**Employment relationship**		
Contract services, temporary contract, commissioned position, self-employed or others	1	1
Statutory public servant or hired public servant	0.86(0.80–0.93)***	0.90(0.83–0.97)**

*p<0.05

**p<0.01

***p<0.001. Model adjusted for Health promotion and intersectoral actions, the participation of OHT in telehealth and instruments and supplies.

*Hosmer Lemeshow* = 0.0833

OHT with consolidated or under development planning of actions showed 1.12 (95% CI: 1.04–1.54) and 1.33 (95% CI: 1.06–1.77) times more chance to present CT/FDA ≥ 1 than those with incipient planning of actions, respectively. The chance of problem-solving capacity was 43% higher in OHT with consolidated integral health. The increase in one unit in the number of supplies was associated with 1.14 times more chance of OHT present CT/FDA ≥ 1. There was more chance of CT/FDA ≥ 1 among OHT that participated in the telehealth and when the municipality had a career plan, and the dentist had complementary training (Table 6).

**Table 6:** Crude and adjusted regression models of the variables associated with ratio between completed treatments and first scheduled dental appointments ≥ 1.

Variables	Crude OR (95% CI)	Adjusted OR (95% CI)
**Work process**		
**Planning of actions**		
Incipient	1	1
Under development	1.28(1.03–1.59)***	1.12(1.04–1.54)*
Consolidated	1.78 (1.45–2.19)***	1.33(1.06–1.77)***
**Health promotion and intersectoral actions**		
Under development	1	
Consolidated	1.64(1.35–1.99)***	
**Integral health care**		
Under development	1	1
Consolidated	1.67(1.46–1.91)***	1.43(1.24–1.65)***
**Participation of OHT in telehealth**		
No	1	1
Yes	1.76(1.46–2.12)***	1.55(1.28–1.87)***
**Receiving students/teachers in teaching activities**		
No	1	
1 to 6 times a week	1.10(0.90–1.35)	
**Number of FHTs in which OHT operates**		
1	1	
2 to 9	0.83(0.68–1.02)	
**Care for individuals outside of OHT coverage area**		
No	1	
Some days of week	1.09(0.89–1.32)	
Every day of week	1.10(0.89–1.35)	
**Structure of OHT**		
**Equipment**	1.15(1.08–1.23)***	
**Supplies**	1.17(1.13–1.21)***	1.14(1.10–1.18)***
**Instruments**	1.17(1.10–1.24)***	
**Professional profile**		
**Work regimen with a career path**		
No	1	1
Yes	1.57(1.30–1.90)***	1.40(1.15–1.70)***
**Complementary dentist training**		
No	1	1
Yes	1.38(1.21–1.58)***	1.26(1.10–1.44)**
**Employment relationship**		
Contract services, temporary contract, commissioned position, self-employed or others	1	
Statutory public servant or hired public servant	1.16(1.02–1.32)*	

*p<0.05

**p<0.01

***p<0.001. Final Model with best fit included only significant variables (p<0.05).

*Hosmer Lemeshow* = 0.0764

## Discussion

The quality of oral health services, considering the performance indicators of access and problem-solving capacity, was favored when positive aspects of the OHT work process and the structure of the health unit were present. Consolidated planning of actions and better structural conditions were associated with a better OHT performance. Variables favorable to the profile of the OHT professionals were positively related to problem-solving capacity but did not favor access to oral health services.

The coverage of the first dental appointment was higher when the planning of actions was consolidated. A systematic review of factors associated with access to oral health in Brazil found that the proper organization of care promotes access [19]. The latent variable "planning of actions" points to important organizational aspects, as it involves working with a periodic agenda, self-evaluations, indicator analysis, varied sources of information, goals, and risk factors. The OHT with consolidated planning of actions plans activities taking into account environmental issues of coverage area (including access to land), biological risks and individual, family and social vulnerabilities (violence, drugs, and others), and local information (demand study, epidemiological scenario, and others). In this way, the OHT includes the economic and social aspects in the planning of actions, which was previously associated with oral health services access [19, 20]. Thus, OHTs that develop these actions not only expand access but also promote equity.

OHTs that cared for individuals outside their coverage area every day of the week had a higher percentage of FDA coverage ≥ mean. Although the establishment of coverage areas is an assumption of the Family Health Strategy, such boundaries should not be barriers to access, and the recognition of social dynamics, the environment, population, and horizontal relationships with other services should cross geographic boundaries [21]. As a social determinant of health, labor is an essential factor related to care outside the coverage area, as one may work in a location that differs from one's place of residence and the health unit where one is registered.

The coverage of the first dental appointment was higher among OHTs that worked with two to nine FHTs. The ratio of an OHT to an FHT is regulated by the Health Ministry and subject to municipal management criteria [22]. Although this proportion favors the coordination and longitudinal aspect of care, a larger population assigned to the OHT may represent a higher number of first scheduled dental appointments. This variable was not associated with problem-solving capacity, which may indicate that the continuity of care was not ensured. A study evaluating oral health indicators identified greater coverage of the first dental appointment with the greater coverage of the FHT [23], which was not investigated in the present investigation and may have influenced the results.

A greater amount of dental equipment favored the offer of first dental appointments. This association has also been reported in previous studies [24, 25]. The ratio between completed treatments and first scheduled dental appointments was also favored as the number of supplies increased. A previous survey considering a minimum set of dental equipment, instruments and supplies found that only 14.8% of OHTs in Brazil can offer the main curative dental procedures in primary care, underscoring the need for structural improvements to ensure quality oral health care [12]. The findings point to the lack of equipment and supplies as limiting factors to access and problem-solving capacity. In Brazil, OHT structure is still a challenge, because around 40% of OHT have not complete supplies and instruments to provide oral health primary care.

Municipalities that had a career path and employed professionals with preserved labor rights were associated with a lower chance of FDA ≥ mean. Human resources in health are recognized as a pillar for strengthening services and ensuring higher quality and access to health [26]. To meet the demands of society, however, adequate numbers of professionals and compliance with labor laws are not enough. The continuing education of health professionals should be in line with the services and based on professional qualifications and the needs of the population [27]. In contrast, problem-solving capacity was more frequent when the municipality had a career path. Analyzing the calculation method of this indicator, if access is negatively impacted by a career plan (denominator), it is reasonable to think that when this factor is present, the CT/FDA ratio tends to increase. Moreover, the precarious hiring of human resources in health, which generates job instability, exerts a negative impact on the creation of personal ties to the job and the reorganization of care practices [10].

There was a dose-response association between planning of actions and CT/FDA ratio comparing OHT with consolidated and under development planning of actions. The planning of actions might favor the problem-solving capacity even though there is some barrier in the work process. The problem-solving capacity benefits from consolidated action planning, as the organization of the agenda, the definition of care goals and the assessment of actions can lead to a patient-centered care model that seeks to solve people's needs. Another aspect is that the problem-solving capacity depends on an efficient flow between primary and secondary care, which is better in context of more organized work process [28].

The problem-solving capacity was also higher among OHT with consolidated integral health, which is those with central regulation available to refer users to dental specialties, those offer specialized consultations available from health network and carry out actions to prevent and detect oral cancer. In Brazil, the achieving of integrality persists as a significant challenge even though the implementation of secondary care in Dental Specialty Centers (CEOs). Previous findings indicate that the presence of the CEOs and greater oral health coverage in the municipalities contribute to integral care, regarding to oral health actions and procedures. National study pointed lower proportions of tooth extractions in municipalities with at least one CEO and with a coverage of greater than 80% by the oral health teams, highlighting that municipalities with a consolidated oral health care network present better performance in the supply of dental care [29]. Although these positive results, there is disparity among states and macro-regions of Brazil in implementation of CEOs [30] and some primary OHTs are located in areas without a Dental Specialty Center, decreasing its problem-solving capacity.

The participation of OHTs in telehealth increased the problem-solving capacity. The telehealth program seeks to strengthen primary care through distance activities that provide interactions among health professionals and offer remote access to diagnostic or even therapeutic support resources. Successful experiences have been reported, including in the field of oral health, and have promoted higher problem-solving capacity and comprehensiveness in care in addition to broadening continuing education and reducing costs [31]. The growth in the use of information and communication technologies in health is a worldwide trend [32] and an important tool for the coordination of care and problem solving [33]. In Brazil, telehealth has contributed to improving the quality of primary care but is still used little by teams in primary care [34].

Complementary dentist training favored problem-solving capacity. A study using data from the PMAQ-AB found a positive association between the educational background of dentists in the field of family health and work process indicators. OHT professionals had a 68% higher frequency of completed treatments and shared the agenda more with other primary care professionals, offered oral health education activities, made more home visits, and used more protocols to define priority actions [35]. A lack of professional qualification is considered an obstacle to good OHT practices in primary care [36].

This nationwide study identified important issues that impact the outcomes of oral health care and service organization. The research included data on structure, process and outcome, favoring scientific evidence and methodological rigor, but also generating some missing data by joining databases. The improvement of data recording and information quality is paramount to the quality of studies. The main limitation of this study regards the methodology used by the PMAQ-AB for the evaluation of OHTs, including voluntary adherence to the program, which may result in selection bias, as the teams that join might be those with better structures and work processes.

Fifteen years after the implementation of the National Oral Health Policy, we identified that structure, and work processes can improve the performance of oral health services. Financial investments in structure may guarantee the instruments, equipment, and supplies to oral health care, enhancing the performance of OHT regarding access and problem-solving capacity. The planning of actions is a powerful way of organizing the work process. It should be motivated and encouraged by local and national managers, thus increasing access and solving the population's oral health needs. There is a need to invest in the specialized care network, which, when available and integrated into primary care, favors OHT's problem-solving capacity. These findings can assist local and national managers in the establishment of economic and social priorities to resources allocation that strengthens the increasing quality of Brazilian oral health care in the public sector.

## Supporting information

S1 Data (XLSX)

S2 Data (XLSX)
